# Association Between Receipt of Cancer Screening and All-Cause Mortality in Older Adults

**DOI:** 10.1001/jamanetworkopen.2021.12062

**Published:** 2021-06-01

**Authors:** Nancy L. Schoenborn, Orla C. Sheehan, David L. Roth, Tansu Cidav, Jin Huang, Shang-En Chung, Talan Zhang, Sei Lee, Qian-Li Xue, Cynthia M. Boyd

**Affiliations:** 1 The Johns Hopkins University School of Medicine, Baltimore, Maryland; 2 The Johns Hopkins Center on Aging and Health, Baltimore, Maryland; 3 Bon Secours Mercy Health St Elizabeth Youngstown Hospital, Youngstown, Ohio; 4 University of California, San Francisco School of Medicine

## Abstract

**Importance:**

Guidelines recommend against routine breast and prostate cancer screenings in older adults with less than 10 years’ life expectancy. One study using a claims-based prognostic index showed that receipt of cancer screening itself was associated with lower mortality, suggesting that the index may misclassify individuals when used to inform cancer screening, but this finding was attributed to residual confounding because the index did not account for functional status.

**Objective:**

To examine whether cancer screening remains significantly associated with all-cause mortality in older adults after accounting for both comorbidities and functional status.

**Design, Setting, and Participants:**

This cohort study included individuals older than 65 years who were eligible for breast or prostate cancer screening and who participated in the 2004 Health and Retirement Study. Data were linked to Medicare claims from 2001 to 2015. Data analysis was conducted from January to November 2020.

**Main Outcomes and Measures:**

A Cox model was used to estimate the association between all-cause mortality over 10 years and receipt of screening mammogram or prostate-specific antigen (PSA) test, adjusting for variables in a prognostic index that included age, sex, comorbidities, and functional status. Potential confounders (ie, education, income, marital status, geographic region, cognition, self-reported health, self-care, and self-perceived mortality risk) of the association between cancer screening and mortality were also tested.

**Results:**

The breast cancer screening cohort included 3257 women (mean [SD] age, 77.8 [7.5] years); the prostate cancer screening cohort included 2085 men (mean [SD] age, 76.1 [6.8] years). Receipt of screening mammogram was associated with lower hazard of all-cause mortality after accounting for all index variables (adjusted hazard ratio [aHR], 0.67; 95% CI, 0.60-0.74). A weaker, but still statistically significant, association was found for screening PSA (aHR 0.88; 95% CI, 0.78-0.99). None of the potential confounders attenuated the association between screening and mortality except for cognition, which attenuated the aHR for mammogram from 0.67 (95% CI, 0.60-0.74) to 0.73 (95% CI, 0.64-0.82) and the aHR for PSA from 0.88 (95% CI, 0.78-0.99) to 0.92 (95% CI, 0.80-1.05), making PSA screening no longer statistically significant.

**Conclusions and Relevance:**

In this study, cognition attenuated the observed association between cancer screening and mortality among older adults. These findings suggest that existing mortality prediction algorithms may be missing important variables that are associated with receipt of cancer screening and long-term mortality. Relying solely on algorithms to determine cancer screening may misclassify individuals as having limited life expectancy and stop screening prematurely. Screening decisions need to be individualized and not solely dependent on life expectancy prediction.

## Key Points

**Question:**

Is receipt of cancer screening independently associated with 10-year mortality after accounting for comorbidities and function in older adults?

**Findings:**

In this cohort study of 5342 patients in the Health and Retirement Study, receipt of breast or prostate cancer screening was associated with a lower hazard of 10-year mortality after adjusting for all variables from a prognostic index that included age, comorbidities, and function.

**Meaning:**

These findings suggest that screening decisions need to be individualized and not solely dependent on mortality prediction.

## Teaser

This cohort study examines whether cancer screening is significantly associated with all-cause mortality in older adults after accounting for comorbidities and functional status.

## Introduction

Cancer screening offers potential benefits of early detection and decreased cancer-related mortality and morbidity, but these benefits are not immediate: they have been shown to have a lag time of approximately 10 years.^1,2,3,4,5,6^ Meanwhile, complications and burdens from cancer screening can occur in the short term.^2,3,4,5,6,7,8,9^ Increasingly, research and clinical practice guidelines recommend that cancer screening decisions take into account a patient’s life expectancy, with the rationale that patients with limited life expectancies may be exposed to the short-term harms of screening when they are unlikely to live long enough to benefit.^2,3,4,5,6,10,11,12,13,14^ Most guidelines specifically mention that clinicians should not routinely screen patients for breast, prostate, or colorectal cancers if they have less than 10 years’ life expectancy.^6,10,11,12,13,14^

Although the concept of using limited life expectancy to inform cancer screening is sound, how to best operationalize this concept in practice is controversial. In our prior work,^15,16^ we found that both primary care clinicians and older adults questioned the accuracy of life expectancy predictions. Currently, a number of prognostic indices have been validated to predict mortality for as long as 10 to 14 years; some are based on health conditions (which we refer to as comorbidities in this article) assessed using administrative data, while others incorporate self-reported comorbidities and functional status.^17,18,19,20,21,22,23^ One study using a claims-only prognostic index^24^ showed that the receipt of cancer screening itself was independently associated with 10-year mortality. In this study by Goodwin et al,^24^ the receipt of cancer screening was independently associated with improved survival, such that some patients who were initially predicted to have less than 10 years’ life expectancy but nonetheless received cancer screening actually had better survival so that their life expectancies were no longer limited. The authors concluded that the specific prognostic index used in the study, which was based solely on age and comorbidity measures in administrative data, did not adequately capture important information, such as functional status, and therefore underestimated life expectancy in those who received screening.^24^ Whether receipt of cancer screening is also independently associated with life expectancy when accounting for both comorbidities and functional status is unknown. This information is important both for improving life expectancy predictions in general and for informing how to use predicted life expectancy in cancer screening decision-making. To address this knowledge gap, we aimed to examine the association between the receipt of breast and prostate cancer screenings and all-cause mortality over 10 years in a population-based cohort study after accounting for all the predictors from a validated 10-year mortality prediction index by Lee et al^18^ that includes self-reported functional measures in addition to age and comorbidities.

## Methods

### Data Source and Study Sample

We drew on data from the Health and Retirement Study (HRS) linked to Medicare claims. HRS is a US-based longitudinal survey, started in 1992, of a nationally representative cohort of approximately 20 000 adults older than 50 years.^25,26^ Biennial interviews collected detailed information from participants regarding their health, participation in daily activities, social environment, and economic circumstances. Race was self-identified by participants as part of the HRS survey. Ethnicity was also assessed in HRS, but it was not used in this study because there is stronger evidence for associations between race and all-cause mortality and between race and cancer screening. The HRS study design and procedures have been described previously.^25,26^ For this study, we used HRS-Medicare linked data from the years 2001 to 2015. We focused on adults aged 65 years or older who responded to the HRS 2004 survey to have at least 10 years of follow-up based on available data through 2015 and used claims data in the 3 years prior to 2004 (2001-2004) to identify eligibility for screening and receipt of screening.

We constructed 2 separate cohorts for breast and prostate cancer screenings. In each cohort, we identified older adults eligible for screening based on prior history (ie, no history of breast or prostate cancer) who were continuously enrolled in fee-for-service Medicare for 36 months prior to their 2004 HRS interview. Specifically, we used the first 12 months of this 36-month period to determine eligibility for screening; we then used the subsequent 24 months to identify receipt of screening tests. Using published algorithms, we identified receipt of screening mammograms and prostate-specific antigen (PSA) tests using claims data in the 24 months before the HRS survey (2002-2004), excluding those ineligible for screening based on claims data from the 12 months prior (2001-2002).^27,28^ Participants for whom we did not have complete information necessary for predicting 10-year mortality risk (detailed in the next section) were excluded (eFigure in the Supplement). We assessed receipt of screening during a 2-year period because recommended intervals for the 2 screening tests ranged from annually to biennially.^7,10,11,14^ Details of screening test identification in the claims data and eligibilities for screening are described in the eAppendix in the Supplement. We followed up each cohort for 10 years after the 2004 HRS interview or until death. This study was approved by a Johns Hopkins School of Medicine institutional review board. The requirement for informed consent was waived, as this project was a retrospective analysis of existing data. This study follows the Strengthening the Reporting of Observational Studies in Epidemiology (STROBE) reporting guideline for cohort studies.

### Mortality Risk Prediction Index

We used a prognostic index previously developed by Lee et al^18^ that estimates 10-year mortality risk and life expectancy. This index was developed and validated in the HRS study. The index incorporates information on both comorbidities and function, is applicable to community-dwelling older adults, and has excellent discrimination, with a C statistic of 0.834. Furthermore, its 10-year time frame is the relevant life expectancy threshold used in clinical practice guidelines for breast and prostate cancer screenings.^18^ The Lee index^18^ (eAppendix in the Supplement) uses 12 items, including age, sex, health status and/or comorbidities (body mass index, diabetes, cancer, lung disease, heart disease, smoking), and functional status (difficulty bathing or showering, difficulty managing money, difficulty walking several blocks, and difficulty pushing and/or pulling large objects).

### Statistical Analysis

The outcome of interest was all-cause mortality during 10-year follow-up after the 2004 HRS interview, using the date of death field in the Medicare enrollment files. Our primary objective was to assess whether receipt of screening mammogram or PSA was independently associated with all-cause mortality after accounting for all the variables in the Lee index.^18^ We conducted parallel analyses for women and men. Using Cox proportional hazards regression models, we estimated the association between receipt of cancer screening and all-cause mortality, adjusting for all the variables in the Lee index as well as race. We included each of the 12 Lee index items as individual variables in the model. This was considered our base model.

Given that prior literature has found that receipt of cancer screening is independently associated with better survival after accounting for age, race, and comorbidities,^24^ we hypothesized that receipt of screening may still be independently associated with better survival in our model, even after accounting for functional status at a significance level of *P* < .05 for 2-sided hypothesis testing. Because neither screening mammogram nor PSA testing has been shown to be associated with reduction in all-cause mortality in randomized clinical trials,^7,8^ such a finding would likely be due to selection bias. Therefore, our secondary objective was to identify other potential confounders to the association between receipt of screening and all-cause mortality. Based on available data elements within the HRS, we tested variables including sociodemographic factors (education, income, marital status, geographic location), other markers of health status (cognitive status, self-reported health, self-perceived mortality risk), and markers of self-care (exercise, receipt of influenza vaccine, and visits to doctors and dentists). Documentation on how each variable was assessed is available through HRS.^26^ We selected these variables because they have been associated with receipt of cancer screening, all-cause mortality, or both.^29,30,31,32,33,34,35,36,37,38^ There are multiple cognitive measures in the HRS data, and we chose the total cognition summary score (range, 0-35; higher scores indicate better cognition) because it was the most inclusive and comprehensive measure. We added each of these variables to the base model one at a time separately to assess whether each variable, when added, led to attenuation of the adjusted hazard ratio (aHR) for screening. All variables that had a significant association with all-cause mortality (ie, *P* < .05) when separately added to the base model were then retained in a full model to assess whether their combination would lead to attenuation of the aHR for screening. We also conducted sensitivity analyses without including race in the model. All analyses were performed using SAS version 9.4 (SAS Institute).

## Results

The final analytic sample included 3257 women in the breast cancer screening cohort and 2085 men in the prostate cancer screening cohort; their characteristics are shown in Table 1. Mean (SD) ages were 77.8 (7.1) years for women and 76.1 (6.8) years for men. Most participants in both cohorts were White individuals (2721 [83.5%] women and 1792 [86.0%] men). In the 2 years prior to 2004, 1544 women received screening mammograms (screening rate, 47.4%), and 1065 men received screening PSAs (screening rate, 51.1%). At the end of 10-year follow up, 1640 women (50.4%) and 1152 men (55.3%) had died.

**Table 1.** Study Cohort Characteristics

Characteristic	Patients, No. (%)	
	Breast cancer screening cohort (n = 3257)	Prostate cancer screening cohort (n = 2085)
Age, y		
65-69	467 (14.3)	367 (17.6)
70-74	844 (25.9)	628 (30.1)
75-79	666 (20.5)	501 (24.0)
80-84	614 (18.9)	328 (15.7)
≥85	666 (20.5)	261 (12.5)
Race		
White	2721 (83.5)	1792 (86.0)
Black	447 (13.7)	239 (11.5)
Other^a^	89 (2.7)	54 (2.6)
Lee index^18^ items, excluding age and sex		
BMI <25	1552 (47.7)	728 (34.9)
Diabetes	591 (18.2)	475 (22.8)
Cancer	387 (11.9)	261 (12.5)
Chronic lung disease requiring oxygen or limiting activity	195 (6.0)	163 (7.8)
Congestive heart failure	301 (9.2)	208 (10.0)
Smoking	244 (7.5)	203 (9.7)
Difficulty bathing	557 (17.1)	214 (10.3)
Difficulty managing money	585 (18.0)	360 (17.3)
Difficulty walking	1678 (51.5)	829 (39.8)
Difficulty pushing and/or pulling weight	1638 (50.3)	573 (27.5)
Census region		
Northeast	490 (15.1)	295 (14.2)
Midwest	960 (29.5)	616 (29.6)
South	1413 (43.4)	902 (43.3)
West	392 (12.0)	271 (13.0)
Education		
<High school	1065 (32.7)	702 (33.7)
High school	1213 (37.2)	649 (31.1)
>High school	979 (30.1)	734 (35.2)
Married or lived with a partner	1263 (38.8)	1582 (75.9)
Income, quartile^b^		
1, Lowest	1417 (43.5)	446 (21.4)
2	988 (30.3)	725 (34.8)
3	582 (17.9)	588 (28.2)
4, Highest	270 (8.3)	326 (15.6)
Medicaid recipient	512 (15.8)	201 (9.7)
In previous 2 y		
Overnight hospital stays, mean (SD), No.	0.7 (2.0)	0.7 (1.2)
Doctor visits, mean (SD), No.	10.6 (12.2)	10.3 (12.2)
Saw dentist	1846 (56.9)	1184 (57.0)
Had influenza vaccine	2418 (74.5)	1617 (77.9)
Engaged in vigorous activities at least once a month	685 (21.1)	711 (34.2)
Engaged in moderate activities at least once a month	1999 (61.4)	1545 (74.1)
Total cognition summary score, mean (SD)^c^	20.9 (5.5)	20.7 (4.9)
Self-reported health		
Excellent	222 (6.8)	170 (8.2)
Very good	801 (24.6)	525 (25.2)
Good	1042 (32.1)	647 (31.0)
Fair	780 (24.0)	476 (22.8)
Poor	406 (12.5)	267 (12.8)
Self-reported >50% chance to live another 10 y	1428 (57.7)	866 (54.5)

Abbreviation: BMI, body mass index (calculated as weight in kilograms divided by height in meters squared).

^a^ Other races included individuals who identified as American Indian or Alaska Native, Asian, or other race/ethnicity.

^b^ The first quartile included incomes less than $18 000 per year; second, $18 000 to less than $35 801; third, $35 801 to less than $70 000; fourth, $70 000 and greater.

^c^ The total cognition summary score ranges from 0 to 35, with higher scores indicating better cognition.

Receipt of screening mammogram was associated with lower hazard for all-cause mortality, with an unadjusted HR of 0.40 (95% CI, 0.36-0.44). Similarly, screening PSA was associated with an unadjusted HR of 0.68 (95% CI, 0.61-0.77). After accounting for all the variables in the Lee index^18^ and for race, receipt of mammogram remained associated with a lower hazard for all-cause mortality with an aHR of 0.67 (95% CI, 0.60-0.74). A less strong but still statistically significant association was found for receipt of PSA screening with an aHR of 0.88 (95% CI, 0.78-0.99) (Table 2).

**Table 2.** Association Between Participant Characteristics and All-Cause Mortality During 10-Year Follow-up in the Base Model

Factor	Adjusted HR (95% CI)	
	Breast cancer screening cohort (n = 3257)	Prostate cancer screening cohort (n = 2085)
Receipt of screening	0.67 (0.60-0.74)	0.88 (0.78-0.99)
Lee index^18^ items, excluding sex		
Age, y		
65-69	1 [Reference]	1 [Reference]
70-74	1.53 (1.23-1.91)	1.44 (1.17-1.79)
75-79	2.02 (1.63-2.52)	2.04 (1.65-2.53)
80-84	3.19 (2.58-3.94)	3.40 (2.72-4.25)
≥85	4.75 (3.84-5.87)	6.44 (5.10-8.13)
BMI <25	1.36 (1.22-1.50)	1.29 (1.14-1.46)
Diabetes	1.45 (1.28-1.64)	1.35 (1.18-1.55)
Cancer	1.31 (1.14-1.51)	1.28 (1.09-1.51)
Chronic lung disease	1.58 (1.33-1.89)	1.65 (1.35-2.01)
Congestive heart failure	1.40 (1.20-1.62)	1.36 (1.14-1.63)
Smoking	1.37 (1.14-1.64)	1.76 (1.45-2.13)
Difficulty bathing	1.72 (1.51-1.96)	1.79 (1.48-2.17)
Difficulty managing money	1.57 (1.39-1.79)	1.46 (1.26-1.71)
Difficulty walking	1.51 (1.34-1.71)	1.69 (1.47-1.94)
Difficulty to pushing and/or pulling weight	1.21 (1.08-1.36)	1.29 (1.11-1.49)
Race		
White	1 [Reference]	1 [Reference]
Black	0.97 (0.84-1.11)	0.94 (0.78-1.13)
Other^a^	0.67 (0.48-0.92)	1.38 (0.98-1.95)

Abbreviations: BMI, body mass index (calculated as weight in kilograms divided by height in meters squared); HR, hazard ratio.

^a^ Other races included individuals who identified as American Indian or Alaska Native, Asian, or other race/ethnicity.

We then added the potential confounders separately to the base model one at a time. The associations between each variable and 10-year all-cause mortality are shown in Table 3 and Table 4 for the women and men, respectively. Variables with a significant association with all-cause mortality (ie, *P* < .05) were then included in a full model. We found that many of the tested variables, including income, marital status, total cognition score, self-reported health, self-perceived mortality risk, exercise, and visits to doctors and dentists were significantly associated with all-cause mortality independent of the variables in the base model (ie, receipt of cancer screening, age, race, and the Lee index items). As shown in Table 5, most potential confounders, when added separately to the base model one at a time, did not attenuate the aHRs between either type of screening and all-cause mortality, with aHR changes of no greater than 0.02. The only exception was the cognition variable. When this was added to the base model, the aHR for screening mammogram was attenuated from 0.67 (95% CI, 0.60-0.74) to 0.73 (95% CI, 0.64-0.82); the aHR for screening PSA was attenuated from 0.88 (95% CI, 0.78-0.99) to 0.92 (95% CI, 0.80-1.05) and no longer statistically significant. In the full model with all the significant variables, the aHR for screening mammogram changed to 0.77 (95% CI, 0.68-0.88) and the aHR for screening PSA changed to 0.93 (95% CI, 0.81-1.06).

**Table 3.** Multivariable Model for All-Cause Mortality During 10-Year Follow-up for the Breast Cancer Screening Cohort^a^

Variable	Variable coefficient when added separately to the base model^b^		Full model^c^	
	HR (95% CI)	*P* value	HR (95% CI)	*P* value
Screening mammogram	NA	NA	0.77 (0.68-0.88)	<.001
Age, y				
65-69	NA	NA	1 [Reference]	NA
70-74	NA	NA	1.65 (1.29-2.11)	<.001
75-79	NA	NA	2.08 (1.62-2.66)	
80-84	NA	NA	3.01 (2.34-3.87)	
≥85	NA	NA	4.16 (3.20-5.41)	
Race				
White	NA	NA	1 [Reference]	NA
Black	NA	NA	0.81 (0.67-0.97)	.01
Other^d^	NA	NA	0.56 (0.37-0.85)	
Lee index^18^ items excluding age and sex				
BMI <25	NA	NA	1.36 (1.21-1.54)	<.001
Diabetes	NA	NA	1.37 (1.18-1.60)	<.001
Cancer	NA	NA	1.30 (1.10-1.53)	.001
Chronic lung disease	NA	NA	1.64 (1.31-2.04)	<.001
Congestive heart failure	NA	NA	1.35 (1.12-1.63)	.001
Smoking	NA	NA	1.41 (1.16-1.72)	.001
Difficulty bathing	NA	NA	1.26 (1.05-1.51)	.01
Difficulty managing money	NA	NA	1.08 (0.90-1.29)	.41
Difficulty walking	NA	NA	1.19 (1.03-1.38)	.02
Difficulty pushing and/or pulling weight	NA	NA	1.09 (0.96-1.24)	.20
Census region				
Northeast	1 [Reference]	NA	NA	NA
Midwest	0.97 (0.83-1.14)	.65	NA	NA
South	1.03 (0.89-1.19)		NA	NA
West	1.08 (0.90-1.30)		NA	NA
Education				
>High school	1 [Reference]	NA	NA	NA
<High school	1.11 (0.98-1.17)	.11	NA	NA
High school	1.14 (1.01-1.30)		NA	NA
Married or lived with a partner	0.86 (0.77-0.97)	.02	0.83 (0.72-0.96)	.01
Income quartile^e^				
1, Lowest	1 [Reference]	NA	1 [Reference]	NA
2	1.06 (0.94-1.19)	.04	1.32 (1.14-1.53)	.01
3	0.84 (0.71-0.99)		1.09 (0.89-1.34)	
4, Highest	0.87 (0.70-1.09)		1.26 (0.96-1.63)	
Has Medicaid	1.05 (0.92-1.20)	.51	NA	NA
In the past 2 y				
Overnight hospital stays	1.01 (1.00-1.22)	.18	NA	NA
Doctor visits	1.01 (1.00-1.01)	.01	1.01 (1.00-1.01)	.01
Saw a dentist	0.83 (0.75-0.92)	<.001	0.87 (0.77-0.99)	.03
Had influenza vaccine	1.10 (0.98-1.23)	.13	NA	NA
Vigorous activities at least once per mo	0.75 (0.64-0.88)	<.001	0.85 (0.72-1.01)	.07
Moderate activities at least once per mo	0.70 (0.62-0.79)	<.001	0.74 (0.65-0.85)	<.001
Total cognition summary score^f^	0.95 (0.94-0.97)	<.001	0.95 (0.94-0.97)	<.001
Self-reported health				
Excellent	1 [Reference]	NA	1 [Reference]	NA
Very good	0.96 (0.73-1.24)	<.001	0.98 (0.74-1.31)	.01
Good	1.26 (0.98-1.63)		1.25 (0.95-1.65)	
Fair	1.41 (1.09-1.84)		1.33 (0.99-1.79)	
Poor	1.48 (1.11-1.96)		1.20 (0.86-1.67)	
Self-reported chance to live another 10 y				
>50% Chance	1 [Reference]	NA	1 [Reference]	NA
<50% Chance	1.15 (1.01-1.31)	<.001	1.08 (0.94-1.24)	.03
Missing	1.53 (1.32-1.78)		1.28 (1.07-1.52)	

Abbreviations: BMI, body mass index (calculated as weight in kilograms divided by height in meters squared); HR, hazard ratio; NA, not applicable.

^a^ For categorical variables that have more than 2 comparison groups, the *P* value is for the overall association of the variable as a whole when added to the model.

^b^ Each potential confounder was added separately one at a time to the base model, which included Lee index^18^ items, receipt of screening, and race.

^c^ All variables that had a significant association with all-cause mortality (*P* < .05) when separately added to the base model were retained in the full model.

^d^ Other races included individuals who identified as American Indian or Alaska Native, Asian, or other race/ethnicity.

^e^ The first quartile included incomes less than $18 000 per year; second, $18 000 to less than $35 801; third, $35 801 to less than $70 000; fourth, $70 000 and greater.

^f^ The total cognition summary score ranges from 0 to 35, with higher scores indicating better cognition.

**Table 4.** Multivariable Model for All-Cause Mortality Over 10-Year Follow-up for the Prostate Cancer Screening Cohort^a^

Variable	Variable coefficient when added separately to the base model^b^		Full model^c^	
	HR (95% CI)	*P* value	HR (95% CI)	*P* value
Screening PSA	NA	NA	0.93 (0.81-1.06)	.27
Age, y				
65-69	NA	NA	1 [Reference]	NA
70-74	NA	NA	1.48 (1.15-1.91)	<.001
75-79	NA	NA	2.06 (1.60-2.65)	
80-84	NA	NA	3.07 (2.36-4.01)	
≥85	NA	NA	5.21 (3.88-7.00)	
Race	NA	NA		
White	NA	NA	1 [Reference]	NA
Black	NA	NA	0.65 (0.51-0.83)	.001
Other^d^	NA	NA	1.13 (0.76-1.68)	
Lee index^18^ items excluding age and sex				
BMI <25	NA	NA	1.30 (1.13-1.50)	.001
Diabetes	NA	NA	1.29 (1.09-1.51)	.003
Cancer	NA	NA	1.46 (1.21-1.77)	<.001
Chronic lung disease	NA	NA	1.59 (1.24-2.04)	<.001
Congestive heart failure	NA	NA	1.25 (1.01-1.55)	.04
Smoking	NA	NA	1.53 (1.22-1.93)	<.001
Difficulty bathing	NA	NA	1.53 (1.19-1.98)	.001
Difficulty managing money	NA	NA	1.08 (0.88-1.33)	.46
Difficulty walking	NA	NA	1.38 (1.17-1.62)	<.001
Difficulty pushing and/or pulling weight	NA	NA	1.19 (1.01-1.42)	.04
Census region				
Northeast	1 [Reference]	NA	NA	NA
Midwest	1.00 (0.83-1.21)	.27	NA	NA
South	1.07 (0.90-1.28)		NA	NA
West	0.89 (0.71-1.12)		NA	NA
Education				
>High school	1 [Reference]	NA	NA	NA
<High school	1.18 (1.02-1.36)	.09	NA	NA
High school	1.11 (0.95-1.29)		NA	NA
Married or lived with a partner	0.85 (0.75-0.97)	.02	0.81 (0.70-0.94)	.01
Income quartile^e^				
1, Lowest	1 [Reference]	.07	NA	NA
2	0.90 (0.77-1.05)	NA	NA	NA
3	0.94 (0.80-1.12)		NA	NA
4, Highest	0.75 (0.60-0.93)		NA	NA
Has Medicaid	1.17 (0.97-1.41)	.11	NA	NA
In past 2 y				
Overnight hospital stays	1.17 (1.12-1.23)	<.001	1.17 (1.10-1.25)	<.001
Doctor visits	1.01 (1.00-1.01)	.02	1.00 (1.00-1.01)	.21
Saw a dentist	0.75 (0.66-0.84)	<.001	0.78 (0.68-0.91)	.01
Had influenza vaccine	0.89 (0.77-1.03)	.11	NA	NA
Vigorous activities at least once per mo	0.78 (0.67-0.90)	.001	0.88 (0.75-1.04)	.13
Moderate activities at least once per mo	0.76 (0.66-0.88)	<.001	0.84 (0.71-1.00)	.05
Total cognition summary score^f^	0.93 (0.92-0.95)	<.001	0.94 (0.93-0.96)	<.001
Self-reported health				
Excellent	1 [Reference]	NA	1 [Reference]	NA
Very good	1.16 (0.88-1.54)	.01	1.07 (0.79-1.47)	.41
Good	1.41 (1.08-1.85)		1.26 (0.93-1.71)	
Fair	1.32 (0.99-1.74)		1.14 (0.82-1.58)	
Poor	1.60 (1.18-2.18)		1.15 (0.79-1.66)	
Self-reported chance to live another 10 y				
>50% Chance	1 [Reference]	NA	1 [Reference]	NA
<50% Chance	1.05 (0.90-1.21)	.01	0.96 (0.82-1.13)	.07
Missing	1.37 (1.16-1.62)		1.25 (0.99-1.57)	

Abbreviations: BMI, body mass index (calculated as weight in kilograms divided by height in meters squared); HR, hazard ratio; NA, not applicable; PSA, prostate-specific antigen test.

^a^ For categorical variables that have more than 2 comparison groups, the *P* value is for the overall association of the variable as a whole when added to the model.

^b^ Each potential confounder was added separately one at a time to the base model, which included Lee index^18^ items, receipt of screening, and race.

^c^ All variables that had a significant association with all-cause mortality (*P* < .05) when separately added to the base model were retained in the full model.

^d^ Other races included individuals who identified as American Indian or Alaska Native, Asian, or other race/ethnicity.

^e^ The first quartile included incomes less than $18 000 per year; second, $18 000 to less than $35 801; third, $35 801 to less than $70 000; fourth, $70 000 and greater.

^f^ The total cognition summary score ranges from 0 to 35, with higher scores indicating better cognition.

**Table 5.** Potential Confounders and the Association Between Receipt of Screening and All-Cause Mortality

Potential confounders added to the base model^a^	Adjusted HR associated with	
	Screening mammogram	Screening PSA
Base model only	0.67 (0.60-0.74)	0.88 (0.78-0.99)
Census region		
Midwest	0.66 (0.59-0.74)	0.88 (0.78-1.00)
South		
West		
Education		
<High school	0.67 (0.60-0.75)	0.89 (0.79-1.01)
High school		
Married or lived with a partner	0.67 (0.60-0.75)	0.89 (0.79-1.00)
Income quartile^b^		
2	0.67 (0.60-0.75)	0.89 (0.79-1.00)
3		
4, Highest		
Has Medicaid	0.66 (0.59-0.74)	0.88 (0.78-0.99)
In past 2 y		
Overnight hospital stays	0.67 (0.60-0.74)	0.89 (0.79-1.00)
Doctor visits	0.67 (0.60-0.76)	0.87 (0.77-0.99)
Saw a dentist	0.69 (0.61-0.77)	0.90 (0.80-1.01)
Had influenza vaccine	0.66 (0.59-0.74)	0.89 (0.79-1.00)
Vigorous activities at least once per mo	0.68 (0.60-0.76)	0.88 (0.78-0.99)
Moderate activities at least once per mo	0.68 (0.61-0.76)	0.88 (0.78-0.99)
Total cognition summary score^c^	0.73 (0.64-0.82)	0.92 (0.80-1.05)
Self-reported health		
Very good	0.67 (0.60-0.75)	0.88 (0.78-0.99)
Good		
Fair		
Poor		
Self-reported chance to live another 10 y		
<50% Chance	0.69 (0.61-0.77)	0.89 (0.79-1.00)
Missing		
All variables with significant association with mortality^d^	0.77 (0.68-0.88)	0.93 (0.81-1.06)

Abbreviations: HR, hazard ratio; PSA, prostate-specific antigen test.

^a^ Each potential confounder was added separately one at a time to the base model, which included Lee index^18^ items, receipt of screening, and race. Adjusted HRs reflect changes when a variable was added; the actual model coefficients associated with each variable appear in Table 3 and Table 4.

^b^ The first quartile (income <$18 000 per year) was the reference group. The second quartile included incomes from $18 000 to less than $35 801; third, $35 801 to less than $70 000; fourth, $70 000 and greater.

^c^ The total cognition summary score ranges from 0 to 35, with higher scores indicating better cognition.

^d^ All variables that had a significant association with all-cause mortality (*P* < .05) when separately added to the base model one at a time were then retained in a full model. Complete model output is included in Table 3 and Table 4.

Results were largely unchanged in sensitivity analyses in which race was not included in the model. The association between receipt of either screening and all-cause mortality did not change in the base model or when the potential confounders were added (eTable in the Supplement).

## Discussion

A number of clinical practice guidelines recommend using life expectancy to inform the decision to continue or stop routine cancer screening in older adults,^6,10,11,12,13,14^ but questions remain on how to operationalize this process to inform these decisions. Our study adds to the existing literature by finding that receipt of 2 different types of screening tests were both independently associated with improved survival over 10 years of follow-up even after accounting for all the variables in commonly used life expectancy prediction indices that include demographic, comorbidity, and functional information. The strengths of the associations found in our study are similar to what was found in the article by Goodwin et al,^24^ which used a different prognostic index. Our findings suggest that existing prediction algorithms, including those that are claims based and those based on self-reported comorbidities and function, likely underestimate life expectancy in patients who subsequently received cancer screening. Our results have several important implications.

Consistent with prior literature, many of the variables we tested were significantly associated with all-cause mortality. This was not surprising given that these variables were selected because of known or suspected associations with all-cause mortality. However, we were surprised that most of these variables, with the exception of cognition, did not attenuate the association between receipt of either type of cancer screening and all-cause mortality when added to the base model. The reasons behind this finding are not clear. The base model, which included age, race, and the Lee index^18^ items that measure health conditions, functional status, and smoking status, already accounted for some of the selection bias between receipt of cancer screening and all-cause mortality; this is supported by our finding that the unadjusted HRs of mammogram and PSA (0.40 and 0.68 respectively) were attenuated to aHRs of 0.67 and 0.88, respectively, in the base model. It is possible that the other variables we tested did not capture different aspects of the selection bias than what were already captured by age, race, and the Lee index^18^ items. We did find that cognition attenuated the association between screening and all-cause mortality to some degree. This is consistent with prior literature that suggests that screening rates decrease as the severity of cognitive impairment increases.^39^ However, screening mammogram was still associated with a significantly reduced hazard for death (aHR, 0.73) even after adjusting for cognition. This suggests that in addition to all the demographic, health, social, and personal characteristics that we examined, there is still residual selection bias between those who do and do not receive cancer screening in terms of mortality risk. In other words, there remains some other quality or qualities that are both associated with receipt of cancer screening and better survival that are not captured in the variables we tested. These qualities appear to be powerful and, interestingly, more prominent for mammogram than for PSA. Given that the mammogram requires a separate appointment for patients to schedule and complete the test, whereas the PSA test is a blood test that may often be performed at the doctor’s office or with other laboratory tests, it is possible that the residual confounders are related to motivation, adherence, or resilience.^40^ These types of information are not well captured or routinely collected. Future research should focus on better identifying, collecting, and measuring such nontraditional risk factors. Such information is critically important to improve and refine prognostication accuracy, for the absence of information cannot be fully compensated by sophisticated statistical or machine learning methods.

Second, in the specific context of cancer screening, using 10-year predicted life expectancy as the sole factor to decide on routine screening is likely overly simplistic. Given that the existing prediction algorithms likely underestimate life expectancy in those patients who subsequently receive screening, older adults who have predicted life expectancies of close to 10 years and elect to continue screening may actually have better prognosis than suggested by the algorithms, and continued screening may still be appropriate; they are at risk of being categorized incorrectly as overscreening in the current paradigm. Lowering the life expectancy threshold to 5 to 7 years, as previously suggested,^41^ may avoid this misclassification. However, the optimal threshold should be identified through direct empirical data. Indeed, there is currently no direct evidence on the health outcomes after screening in patients with limited predicted life expectancies, as defined by the existing prognostic algorithms, or how the benefits and harms vary by different levels of predicted life expectancy. This is an important area to examine in future studies. In addition, our results highlight that while prediction algorithms can be valuable tools to inform cancer screening considerations and discussions, the final decision still needs to be tailored to the individual as part of shared decision-making between the clinician and patient because the clinician and the patient likely have access to information not captured in the prediction algorithms and have a deeper understanding of the patient’s prognosis than indices can predict.

### Limitations

Our study has a number of limitations. We required that participants have continuous Medicare fee-for-service coverage in the 3 years before 2004 to assess receipt of screening, and therefore, the study cohort may not be generalizable to older adults covered by Medicare Advantage plans. We used a published algorithm to identify screening tests in the claims data, but claims can be susceptible to coding and measurement errors.^27,28^ The study design did not allow for examination of the role of patient preference in screening decisions. Although the overall study cohorts were large, the sample sizes in certain subgroups were smaller, leading to relatively wide confidence intervals for some of our estimates. We used a specific prognostic index by Lee et al^18^; future studies may assess whether similar results can be replicated using different prognostic indices in different populations. When examining potential confounders for the association of receipt of screening with all-cause mortality, we focused on patient characteristics and did not examine clinician characteristics. We also were not able to test other patient characteristics that may confound the association between receipt of cancer screening and mortality, such as social isolation and health care access, which can be explored in future studies.

## Conclusions

In this cohort study, we found that receipt of breast or prostate cancer screenings was associated with lower hazard of all-cause mortality after accounting for age, comorbidities, and functional status. Existing prediction algorithms may be missing important variables that are associated with cancer screening and long-term mortality. Screening decisions need to be individualized and not solely dependent on life expectancy prediction.

## Notes

Supplement. **eAppendix.** Supplemental Methods **eFigure.** Flow Diagram of the 2 Study Cohorts **eTable.** Sensitivity Analyses
