# Supplementary material for: Association Between Receipt of Cancer Screening and All-Cause Mortality in Older Adults
Source: JAMA Netw Open. 2021 Jun 1;4(6):e2112062. doi: 10.1001/jamanetworkopen.2021.12062 (PMC8170538; doi:10.1001/jamanetworkopen.2021.12062)
Supplement: Supplement. — eAppendix. Supplemental Methods eFigure. Flow Diagram of the 2 Study Cohorts eTable. Sensitivity Analyses [file jamanetwopen-e2112062-s001.pdf]

Supplemental Online Content

Schoenborn NL, Sheehan OC, Roth DL, et al. Association between receipt of cancer screening and all-cause mortality in older adults. *JAMA Netw Open*. 2021;4(6):e2112062. doi:10.1001/jamanetworkopen.2021.12062

eAppendix. Supplemental Methods

eFigure. Flow Diagram of the 2 Study Cohorts

eTable. Sensitivity Analyses

This supplemental material has been provided by the authors to give readers additional information about their work.

© 2021 Schoenborn NL et al. *JAMA Network Open.*

eAppendix. Supplemental Methods

Algorithms used to identify breast and prostate cancer screening tests in Medicare claims data.

We used claims data in the 24 months before the HRS survey (2002-2004) to identify the screening tests and used claims from the 12 months prior (2001-2002) to assess for eligibility for screening.

Screening mammograms

We used a three-step algorithm to distinguish screening from diagnostic mammograms validated by Fenton et al.[28]

1) Include if mammography was for screening (76092, 77057, G0202, any GG modifier, G0203/05); Exclude if mammography was for diagnosis (76090, 76091, 77055, 77056, G0204, G0206);

2) Exclude mammography if the woman has already received mammography in the prior 9 months;

3) Exclude if any ICD-9 code for breast cancer in the previous year (174x, 233.0, V103, 611.72);

Screening prostate-specific antigen (PSA) test

We used the algorithm used by Walter et al.[27]

PSA test: CPT code G0103 and 84153.

We excluded men who were ineligible for screening due to prior history in the previous year. Further, a PSA test is considered a screening test only if there are no relevant symptom codes in the 3 months prior to avoid diagnostic testing.

Prior history:
- Prostate cancer (ICD-9 185, V1046)
- Prostatectomy (ICD-9 60.21, 60.29, 60.3-60.6, 60.61, 60.62, 60.69, CPT 55810, 55812, 55815, 55801, 55821, 55831, 55842, 55845)
- Androgen deprivation therapy (CPT J1950, J9202, J9217, J9218, J9219)
- History of elevated PSA (ICD-9 790.93)

Symptoms:
- Urinary obstruction (ICD-9 599.6)
- Hematuria (ICD-9 599.7)
- Prostatitis (ICD-9 601-601.9)
- Other disorders of the prostate (ICD-9 602-602.9)
- Unexplained weight loss (ICD-9 783.21)
- Back pain (ICD-9 724.5)

The 12 items included in the prognostic index developed and validated by Lee et al.[18]

Index items	
Age	60-64
	65-69
	70-74
	75-79
	80-84
	≥ 85
Male sex	
Body mass index<25	
Diabetes	
Cancer	
Chronic lung disease that limits activities or requires oxygen	
Congestive heart failure	
Smoking	
Difficulty with bathing or showering	
Difficulty managing money	
Difficulty walking several blocks	
Difficulty with pulling or pushing large objects	

© 2021 Schoenborn NL et al. *JAMA Network Open.*

eFigure. Flow Diagram of the 2 Study Cohorts

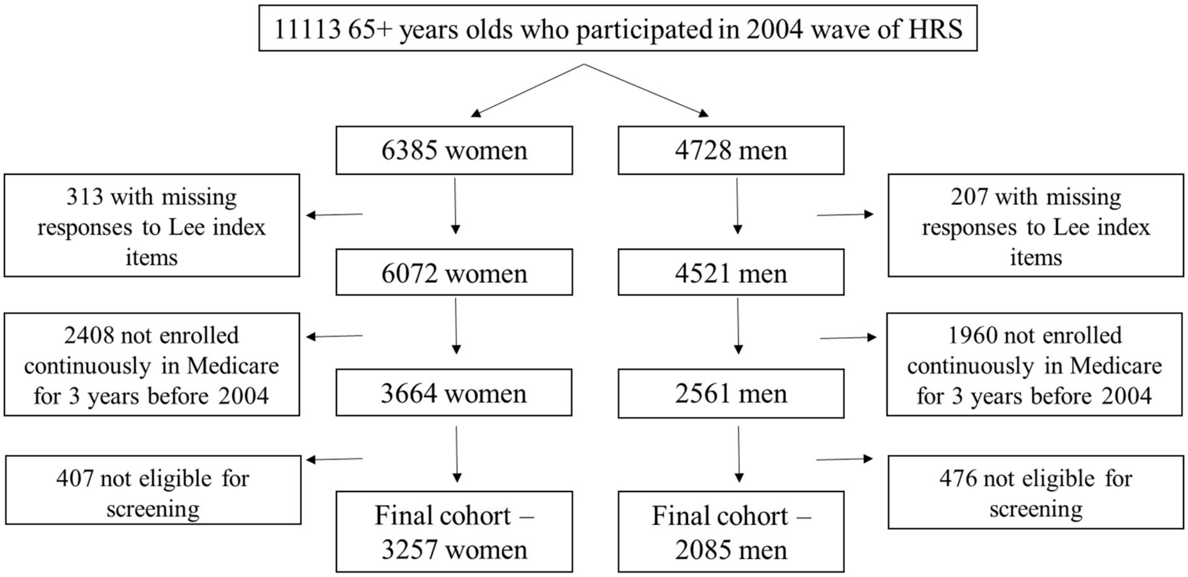

© 2021 Schoenborn NL et al. *JAMA Network Open.*

eTable. Sensitivity Analyses

eTable A. Association between participant characteristics and all-cause mortality over 10-year follow up in the "base model" that included receipt of screening, Lee index items, without race.

	Breast cancer screening (n=3257)	Prostate cancer screening (n=2085)
	Adjusted HR (95% CI)	Adjusted HR (95% CI)
Receipt of screening	0.67 (0.60, 0.74)	0.88 (0.78, 0.99)
Lee index items excluding sex<br>Age		
70-74 vs. 65-69	1.53 (1.23, 1.90)	1.46 (1.18, 1.80)
75-79 vs. 65-69	2.03 (1.63, 2.52)	2.07 (1.67, 2.56)
80-84 vs. 65-69	3.22 (2.61, 3.97)	3.42 (2.74, 4.28)
85+ vs. 65-69	4.77 (3.86, 5.89)	6.47 (5.13, 8.17)
BMI < 25	1.36 (1.23, 1.51)	1.29 (1.14, 1.45)
Diabetes	1.43 (1.26, 1.61)	1.34 (1.17, 1.54)
Cancer	1.32 (1.15, 1.52)	1.28 (1.09, 1.51)
Lung disease	1.60 (1.34, 1.90)	1.65 (1.35, 2.01)
Heart disease	1.40 (1.21, 1.62)	1.36 (1.14, 1.63)
Smoking	1.35 (1.13, 1.62)	1.75 (1.45, 2.13)
Difficulty to bath	1.72 (1.51, 1.96)	1.78 (1.47, 2.15)
Difficulty to manage money	1.56 (1.37, 1.77)	1.45 (1.24, 1.69)
Difficulty to walk	1.50 (1.33, 1.70)	1.69 (1.47, 1.94)
Difficulty to push weight	1.21 (1.08, 1.36)	1.29 (1.11, 1.49)

© 2021 Schoenborn NL et al. *JAMA Network Open.*

eTable B. Multivariable model for all-cause mortality over 10-year follow up for the breast cancer screening cohort without including race in the model. [a]

	Variable coefficient when added separately to the "base model" [b]		Full model [c]	
Variable	HR (95% CI)	p-value	HR (95% CI)	p-value
Screening mammogram	NA	NA	0.77 (0.68, 0.87)	<0.01
Age	NA	NA		
70-74 vs. 65-69			1.66 (1.30, 2.12)	<0.01
75-79 vs. 65-69			2.11 (1.65, 2.70)	
80-84 vs. 65-69			3.13 (2.44, 4.02)	
85+ vs. 65-69			4.33 (3.34, 5.62)	
Lee index items excluding age, sex	NA	NA		
BMI < 25			1.38 (1.22, 1.55)	<0.01
Diabetes			1.33 (1.15, 1.55)	<0.01
Cancer			1.31 (1.11 ,1.54)	<0.01
Chronic lung disease			1.69 (1.36, 2.10)	<0.01
Congestive heart failure			1.35 (1.12, 1.63)	<0.01
Smoking			1.38 (1.14, 1.69)	<0.01
Difficulty to bath			1.25 (1.05, 1.50)	0.01
Difficulty to manage money			1.10 (0.92, 1.30)	0.41
Difficulty to walk			1.19 (1.03, 1.38)	0.02
Difficulty to push weight			1.09 (0.96, 1.24)	0.20
Census region, (ref=Northeast)				
Midwest	0.98 (0.84, 1.14)	0.74	/	/
South	1.02 (0.88, 1.18)			
West	1.07 (0.89, 1.29)			
Education, (ref= > High school)				
< High school	1.09 (0.96, 1.24)	0.12	/	/
High school	1.14 (1.01, 1.30)			
Married or lived with a partner	0.87 (0.77, 0.98)	0.02	0.83 (0.72, 0.97)	0.02
Income, (ref= lowest quartile)				
2nd quartile	1.07 (0.96, 1.21)	0.04	1.34 (1.16, 1.56)	<0.01
3rd quartile	0.85 (0.73, 1.00)		1.11 (0.91, 1.36)	
Highest quartile	0.89 (0.71, 1.11)		1.27 (0.98, 1.65)	
Has Medicaid	1.02 (0.89, 1.16)	0.81	/	/
# of overnight hospital stays	1.01 (1.00, 1.22)	0.17	/	/
# of doctor visit [c]	1.01 (1.00, 1.01)	0.01	1.01 (1.00, 1.01)	0.01
Having seen a dentist [c]	0.84 (0.76, 0.93)	<0.01	0.88 (0.77, 0.99)	0.04
Has had flu shot	1.10 (0.98, 1.23)	0.11	/	/
Vigorous activities at least 1x/month	0.75 (0.64, 0.88)	<0.01	0.86 (0.72, 1.02)	0.09
Moderate activities at least 1x/month	0.70 (0.62, 0.79)	<0.01	0.75 (0.65, 0.86)	<0.01
Total cognition summary score	0.96 (0.95, 0.97)	<0.01	0.96 (0.95, 0.97)	<0.01
Self-reported health, (ref=excellent)				
Very good	0.95 (0.73, 1.24)	<0.01	0.97 (0.73, 1.29)	0.02
Good	1.25 (0.97, 1.61)		1.24 (0.94, 1.64)	
Fair	1.39 (1.07, 1.81)		1.30 (0.97, 1.74)	
Poor	1.46 (1.10, 1.93)		1.18 (0.84, 1.64)	

Self-reported chance to live another 10 years, (ref = >50% chance)				
<50% chance	1.15 (1.01, 1.31)	<0.01	1.09 (0.95, 1.25)	0.04
Missing	1.50 (1.92, 1.74)		1.25 (1.06, 1.50)	

[a] For categorical variables that have more than two comparison groups, the p-value is for the overall effect of the variable as a whole when added to the model.

[b] Each potential confounder was added separately one at a time to the “base model”. “Base model” included Lee index items, receipt of screening but not race in this analysis.

[c] All variables that had a significant association with all-cause mortality (p<0.05) when separately added to the “base model” were then retained in the full model.

[d] In the previous 2 years.

© 2021 Schoenborn NL et al. *JAMA Network Open.*

eTable C. Multivariable model for all-cause mortality over 10-year follow up for the prostate cancer screening cohort without including race in the model. [a]

	Variable coefficient when added separately to the "base model" [b]		Full model [c]	
Variable	HR (95% CI)	p-value	HR (95% CI)	p-value
Screening PSA	NA	NA	0.92 (0.80, 1.06)	0.24
Age	NA	NA		
70-74 vs. 65-69			1.51 (1.18, 1.94)	<0.01
75-79 vs. 65-69			2.12 (1.65, 2.73)	
80-84 vs. 65-69			3.19 (2.45, 4.16)	
85+ vs. 65-69			5.46 (4.07, 7.32)	
Lee index items excluding age, sex	NA	NA		
BMI < 25			1.30 (1.13, 1.50)	<0.01
Diabetes			1.26 (1.07, 1.48)	0.01
Cancer			1.44 (1.19, 1.75)	<0.01
Chronic lung disease			1.66 (1.30, 2.12)	<0.01
Congestive heart failure			1.30 (1.05, 1.61)	0.02
Smoking			1.55 (1.23, 1.94)	<0.01
Difficulty to bath			1.53 (1.19, 1.97)	<0.01
Difficulty to manage money			1.09 (0.89, 1.34)	0.40
Difficulty to walk			1.38 (1.17, 1.62)	<0.01
Difficulty to push weight			1.17 (0.98, 1.39)	0.08
Census region, (ref=Northeast)				
Midwest	0.99 (0.82, 1.20)	0.35	/	/
South	1.06 (0.89, 1.27)			
West	0.89 (0.71, 1.12)			
Education, (ref= > High school)				
< High school	1.17 (1.01, 1.35)	0.11	/	/
High school	1.10 (0.95, 1.29)			
Married or lived with a partner	0.85 (0.74, 0.97)	0.02	0.82 (0.70, 0.95)	0.01
Income, (ref= lowest quartile)				
2nd quartile	0.90 (0.78, 1.05)	0.07	/	/
3rd quartile	0.94 (0.80, 1.11)			
Highest quartile	0.75 (0.60, 0.93)			
Has Medicaid	1.18 (0.98, 1.42)	0.09	/	/
# of overnight hospital stays	1.17 (1.12, 1.23)	<0.01	1.16 (1.09, 1.24)	<0.01
# of doctor visit [c]	1.01 (1.00, 1.01)	0.02	1.00 (1.00, 1.01)	0.24
Having seen a dentist [c]	0.75 (0.66, 0.84)	<0.01	0.80 (0.69, 0.92)	<0.01
Has had flu shot	0.90 (0.78, 1.04)	0.15	/	/
Vigorous activities at least 1x/month	0.78 (0.67, 0.90)	<0.01	0.88 (0.75, 1.04)	0.13
Moderate activities at least 1x/month	0.77 (0.66, 0.89)	<0.01	0.86 (0.73, 1.02)	0.09
Total cognition summary score	0.94 (0.92, 0.95)	<0.01	0.95 (0.93, 0.96)	<0.01
Self-reported health, (ref=excellent)				
Very good	1.16 (0.88, 1.54)	<0.01	1.05 (0.77, 1.43)	0.36
Good	1.42 (1.08, 1.86)		1.24 (0.92, 1.69)	
Fair	1.32 (1.00, 1.75)		1.10 (0.79, 1.52)	
Poor	1.62 (1.20, 2.20)		1.12 (0.77, 1.62)	

© 2021 Schoenborn NL et al. *JAMA Network Open.*

Self-reported chance to live another 10 years, (ref = >50% chance)				
<50% chance	1.05 (0.91, 1.62)	<0.01	1.01 (0.86, 1.18)	0.13
Missing	1.37 (1.17, 1.62)		1.24 (0.99, 1.57)	

[a] For categorical variables that have more than two comparison groups, the p-value is for the overall effect of the variable as a whole when added to the model.

[b] Each potential confounder was added separately one at a time to the "base model". "Base model" included Lee index items, receipt of screening but not race in this analysis.

[c] All variables that had a significant association with all-cause mortality (p<0.05) when separately added to the "base model" were then retained in the full model.

[d] In the previous 2 years.

© 2021 Schoenborn NL et al. *JAMA Network Open.*

eTable D. The effect of potential confounders on the association between receipt of screening and all-cause mortality without including race in the model.

Potential confounders added to the base model [a]	Adjusted HR associated with **screening mammogram**	Adjusted HR associated with **screening PSA**
Base model only	0.67 (0.60, 0.74)	0.88 (0.78, 0.99)
Census region, (ref = Northeast)<br>Midwest<br>South<br>West	0.66 (0.60,0.74)	0.88 (0.78, 1.00)
Education, (ref= > high school)<br>< High school<br>High school	0.67 (0.60, 0.75)	0.89 (0.79, 1.01)
Married or lived with a partner	0.67 (0.60, 0.75)	0.89 (0.79, 1.00)
Income, (ref= lowest quartile)<br>2nd quartile<br>3rd quartile<br>Highest quartile	0.67 (0.60, 0.75)	0.89 (0.79, 1.00)
Has Medicaid	0.66 (0.59, 0.74)	0.88 (0.78, 0.99)
# of overnight hospital stays, [b]	0.67 (0.60, 0.74)	0.89 (0.79, 1.00)
# of doctor visit, [b]	0.68 (0.60, 0.76)	0.87 (0.77, 0.98)
Having seen a dentist [b]	0.69 (0.61, 0.77)	0.90 (0.80, 1.01)
Has had flu shot [b]	0.66 (0.59, 0.74)	0.89 (0.79, 1.00)
Vigorous activities at least once a month	0.68 (0.61, 0.76)	0.88 (0.78, 0.99)
Moderate activities at least once a month	0.68 (0.61, 0.76)	0.88 (0.78, 0.99)
Total cognition summary score	0.73 (0.65, 0.82)	0.91 (0.80, 1.04)
Self-reported health, (ref=excellent)<br>Very good<br>Good<br>Fair<br>Poor	0.67 (0.60, 0.75)	0.88 (0.78, 0.99)
Self-reported chance to live another 10 years, (ref = >50% chance)<br><50% chance<br>Missing	0.69 (0.61, 0.77)	0.90 (0.79, 1.00)
All variables with significant association with mortality [c]	0.77 (0.68, 0.87)	0.92 (0.80, 1.06)

[a] Each potential confounder was added separately one at a time to the "base model". "Base model" included Lee index items, receipt of screening but not race in this analysis. Here we show how the adjusted HR for screening changes when a variable is added, the actual model coefficients associated with each variable are included in Supplemental Tables 1b and 1c.
[b] In the previous 2 years.
[c] All variables that had a significant association with all-cause mortality ($p<0.05$) when separately added to the "base model" one at a time were then retained in a full model. Complete model output is included in Supplemental Tables 1b and 1c.
